# Vegetables and Their Bioactive Compounds as Anti-Aging Drugs

**DOI:** 10.3390/molecules27072316

**Published:** 2022-04-02

**Authors:** Hamza Mechchate, Aicha El Allam, Nasreddine El Omari, Naoufal El Hachlafi, Mohammad Ali Shariati, Polrat Wilairatana, Mohammad S. Mubarak, Abdelhakim Bouyahya

**Affiliations:** 1 Laboratory of Biotechnology, Environment, Agri-Food and Health (LBEAS), Faculty of Sciences, University Sidi Mohamed Ben Abdellah (USMBA), Fez B.P. 1796, Morocco; hamza.mechchate@usmba.ac.ma; 2 Laboratory of Human Pathologies Biology, Department of Biology, Faculty of Sciences, Mohammed V University in Rabat, Rabat 10106, Morocco; elallamaicha@gmail.com; 3 Laboratory of Histology, Embryology and Cytogenetic, Faculty of Medicine and Pharmacy, Mohammed V University in Rabat, Rabat 10100, Morocco; nasrelomari@gmail.com; 4 Microbial Biotechnology and Bioactive Molecules Laboratory, Sciences and Technologies Faculty, Sidi Mohmed Ben Abdellah University, Imouzzer Road, Fez P.O. Box 2002, Morocco; naoufal.elhachlafi@usmba.ac.ma; 5 Department of Scientific Research, K.G. Razumovsky Moscow State University of Technologies and Management (The First Cossack University), 73, Zemlyanoy Val St., 109004 Moscow, Russia; shariatymohammadali@gmail.com; 6 Department of Clinical Tropical Medicine, Faculty of Tropical Medicine, Mahidol University, Bangkok 10400, Thailand; 7 Department of Chemistry, The University of Jordan, Amman 11942, Jordan

**Keywords:** aging, senescence, wrinkles, vegetables, natural compounds, anti-aging activity, apoptosis, senescence

## Abstract

Aging is a continuous process over time that is mainly related to natural alterations in mechanical–biological processes. This phenomenon is due to several factors, including the time and energy of biological processes. Aging can be attributed to biological factors such as oxidative stress, cell longevity, and stem cell senescence. Currently, aging is associated with several diseases, such as neurodegenerative diseases, cancer, and other diseases related to oxidative stress. In addition, certain natural molecules, including those derived from vegetables, have shown the ability to delay the aging process. Their effects are linked to different mechanisms of action, such as tissue regeneration and the activation of longevity and anti-senescence genes. The present work discusses the impact of vegetables, and bioactive compounds isolated from vegetables, against the physiological and pathological aging process and accompanying human diseases.

## 1. Introduction

Higher organisms are organized into organs and tissues with complex interconnections. During the life of a higher organism, the loss of functional and structural cells is directly compensated for by stem cells. However, over time, the ability of these stem cells to generate newly differentiated cells weakens for two reasons: the depletion of stem cells and the reduction in the energy necessary for the survival of these cells (ΔG), which is lost in the form of ΔS [1,2,3,4]. This mechanical–biological process results in aging, which is essentially associated with the senescence of the cells constituting the organism (their inability to divide and the inability of cells to be renewed). Thus, this process is becoming more important with the increase in life expectancy leading to the appearance of a certain number of so-called age-related diseases. Several recent works have deciphered the molecular nature of certain pathologies. They have demonstrated a close relationship between the aging process and several human pathologies, particularly cancer, Alzheimer’s, and Parkinson’s diseases [5,6,7].

Recently, specific investigations have suggested that using natural (biological) resources could effectively delay physiological and pathological aging, and thus prevent the appearance of these pathologies [1,2,8]. Indeed, nature is full of natural products, including those isolated and derived from vegetables, characterized by the synthesis of secondary metabolites. Secondary metabolites include several chemical families, mainly flavonoids, phenolic acids, and terpenoids [9,10,11,12,13,14]. Additionally, other substances belonging to the primary metabolites have also shown significant effects against aging. These molecules (secondary and primary metabolites) and their active ingredients are mainly mediated by specific mechanisms such as tissue generation, telomere activation, anti-senescence action, DNA repair, and targeted antioxidant activity [15,16]. Accordingly, the present work highlights all the factors inducing physiological and pathological aging, and discusses the effects of vegetables and their bioactive substances against the various mechanisms causing this phenomenon.

## 2. Aging and Its Molecular Mechanism

Human aging, unlike sickness, is a progressive time-related process. It varies from person to person, and corresponds biologically to a loss of homeostasis, an increase in the organism’s sensitivity and susceptibility to disease and death, and the progressive degeneration of cells, tissues, and organs associated with advancing age [17]. These aspects of deterioration are called senescence, and are responsible for the weakening of an individual’s health. They also cause physiological changes in ‘‘regular’’ aging, such as menopause and decreased kidney function, and age-related disorders, such as coronary heart diseases, in ‘‘ordinary’’ aging. Several variables contribute to the loss of homeostasis, which is ultimately the consequence of a genetic program; some models propose that genes function to increase or decrease the relative risk of death by increasing the likelihood of disease [17]. Changes in the crystal structure or macromolecular aggregation at the molecular level, the loss and shortening of telomeres at the chromosomal level, changes in mitochondria, and the accumulation of lipofuscins inducing cellular aging are all monitored as part of the aging process [17], along with the appearance of cross-linking lesions of collagen and elastic fibers, and amyloid deposition. This usually causes a change in an organism’s appearance, function, and behavior [17,18].

The human average life duration has risen substantially over time; the most remarkable possible lifespan has remained stable, ranging from 90 to 100 years, and differs from person to person. The average human life expectancy has increased in recent years due to changes and developments in disease management and the socio-economic status and nutritional status of individuals, as well as fewer accidents; the improvement in all these factors has contributed to an increase in the average human life expectancy [19]. At the very least, increased mortality after maturation [20,21,22], changes in the biochemical composition of tissues [23], progressive declines in physiological capacity [24,25,26], and reduced abilities to respond adaptively to environmental stimuli are among the numerous patterns associated with aging in mammals. However, these are as yet unclear and unverified, because the mechanisms of aging could be highly different between animals, tissues, and cells.

**Figure 1:**
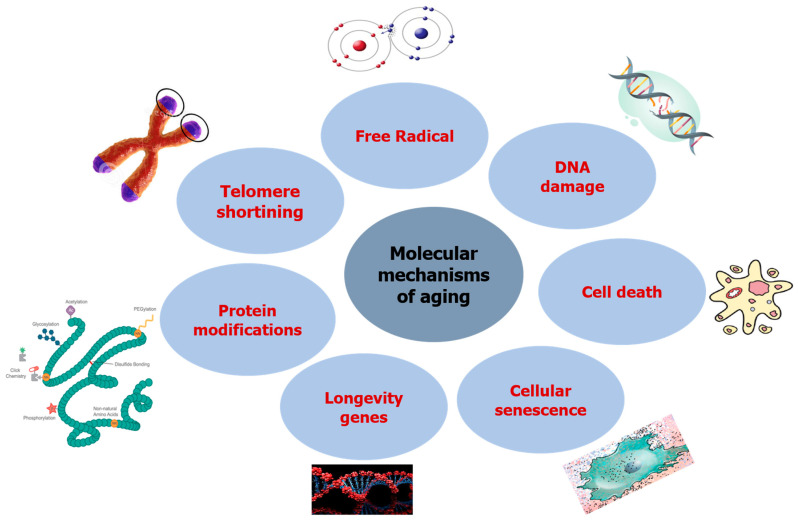
Molecular mechanisms inducing aging.

Aging mechanisms in humans differ significantly from those of one organism, tissue, or cell, making it impossible to establish a specific mechanism. On the other hand, other academics have focused on understanding the evolutionary foundation of senescence in the aging mechanism so as to understand the aging process and study the various genes involved in senescence, which can be divided into three groups; namely, genes regulating somatic maintenance and repair, genes promoting early survival, and genes causing late deleterious mutations [27,28]. These essential genes have been shown to impact the evolution and survival of a species, and may play a role in reducing longevity due to higher growth and repeatability. The published research in molecular genetics has revealed that cellular senescence could have an opposite pleiotropic effect, preventing cancer while simultaneously contributing to organism aging. Figure 1 describes the molecular mechanisms involved in the aging process.

### 2.1. DNA Damage

The molecular mechanisms of aging are still unknown. According to researchers, aging is simply a physiological decline caused by the accumulation of random damage to vital molecules in aging populations. It is an example of background radiation causing genetic damage that leads to mutations, resulting in functional impairment and death [29,30]. The primary example of this theory is the ability to repair DNA damage caused by intrinsic sources such as replication defects and chemical modifications of DNA [31,32], or by external sources, including UV and genotoxic drugs [31,32] in species with different lifespans [33]. Mutations, transcription, replication halts, and the DNA damage response are triggered when DNA is damaged (DDR). These DDRs block cell cycle progression and activate signaling pathways affecting the cell via repair, apoptosis, or cellular senescence [34]. In addition, the presence of defects in genes involved in the DNA repair system causes the accumulation of unrepaired DNA and chromosomal damage [35].

On the other hand, exonuclease 1 (EXO1) and postmeiotic segregation increased 2 (PMS2) are two components that play a vital role in the DNA repair system. Their function contributes to human longevity [36,37]. Indeed, various investigations in human patients and mouse models have highlighted the relevance of DNA repair in the aging process [38]. Thus, it is not the DNA damage that causes aging; instead, it is about how the cell reacts to DNA damage and how this response will affect the organism’s life. In this respect, any abnormality or mutation in DNA repair pathways—such as Werner’s syndrome caused by a mutation in Werner’s syndrome protein (WRN), a gene that encodes a RecQ DNA helicase essential for replication stress management and telomere stability [34,39]—causes rapid aging and shorter lifespan [40]. Furthermore, researchers discovered several clinical disorders caused by problems with the genome maintenance system and aging-related syndromes. For example, Cockayne syndrome is affected by transcription-coupled nucleotide excision repair (TC-NER) [18,41]—these DNA damage repairs are generally induced after the exposure of genetic material to UV rays; ataxia-telangiectasia (AT) affected by DNA damage response—an immune deficiency affecting the humoral pathway and manifested by progressive cerebellar ataxia, linked to genetic instability of the genes that code for a protein kinase (coded by the MRE11 gene) controlling the repair of DNA double-strand breaks, especially in cerebellar and endothelial cells; Werner syndrome impacted by telomere maintenance and replication stress [17,42]—an autosomal recessive genetic disease inducing premature aging due to genetic instability, which affects the DNA repair system; Rothmund–Thomson syndrome, affected by DNA replication starting codes [43,44]—a hereditary genetic dermatosis, manifesting in dermatological signs linked to premature aging, which predisposes one to skin cancer.

### 2.2. Free Radicals

It has been discovered that the majority of age-related alterations are caused by molecular damage induced by free radical [45,46,47] atoms or molecules possessing an unpaired and reactive electron, constituting another possible cause of aging. These oxygen-derived species can react with macromolecules to produce free radicals from the attacked molecules [47,48], and act as secondary messengers in signaling pathways implicated in the control of various mechanisms, such as changes in gene expression, cell replication, differentiation, and apoptotic cell death [49,50,51]. The generation of these free radicals in human organs such as the heart, kidney, and liver affects maximal lifespan [52,53]. In this context, nutritional antioxidants have been found to reduce the risk of vascular dementia, heart disease, and cancer in humans [54,55]. Reactive oxygen species (ROS), in turn, play a role in the somatic accumulation of mutations in mitochondrial DNA, which is one of the developmental–genetic aspects of aging. These mutations result in a gradual loss of bioenergetic capacity, as well as aging and cell death [56,57,58]—‘‘Mitochondrial aging’s redox mechanism’’ [59]. As people age, oxygenated free radicals play a role in mitochondrial DNA (mtDNA) damage [60,61,62]. This damage causes inefficient mitochondrial respiration, which increases with age, as well as the onset of age-related illnesses, including Parkinson’s disease [63,64], Alzheimer’s disease [65,66], Huntington’s chorea [67,68], and others. mtDNA is passed down through the generations and replicates. Moreover, it is much more susceptible to mutations than nuclear DNA, and the rate of mutations rises with age [69,70].

### 2.3. Telomere Shortening

In addition to DNA damage, telomere length is associated with age-related disorders, whereby telomere shortening can impair somatic stem cell function. Findings indicate that telomerase-deficient mice have short telomeres and age prematurely, whereas cancer-resistant mice with high telomerase expression have long telomeres and age more slowly [71,72]. The phenomenon of telomere shortening also remains a feature and a counting mechanism of senescent cells [73,74]. Telomeres consist of long stretches of TTAGGG repeats located at the ends of chromosomes, and act as protectors that prevent them from degrading or fusing with other chromosome ends [60]. Telomerase expression is limited in human somatic cells, leading to telomere reduction and replicative senescence [61]. In fibroblasts and peripheral blood lymphocytes, the average length of the terminal restriction fragment of chromosomes decreases with age [75,76,77]. These findings show that telomere length, rather than telomerase activity, is the most critical determinant in cellular aging. Furthermore, the shortest telomere affects cell viability and chromosome stability, rather than the average-length telomeres. In this respect, several studies have demonstrated that in aged animals and humans, telomeres shorten over time [71,78]. Shorter telomeres are associated with an increased risk of death [79] and replicative senescence; they prevent cancer cells from dividing indefinitely [80]. In contrast, non-enzymatic telomere elongation extends cell lifetime in the laboratory [81].

### 2.4. Protein Modifications

Proteins are the building blocks of living creatures’ cellular and physiological functions, and their physical and chemical qualities determine their activities and functions. Protein folding and final conformation, and biochemical activity, stability, and half-life are all affected by the primary sequence [82]. Researchers have shown that protein repair and modification might play a role in longevity in certain situations [83]. The oxidation of amino acid residues, metal-catalyzed oxidation, and change caused by lipid oxidation products reduce the specific activity of numerous enzymes, affect thermal stability, and increase the carbonyl content of proteins [84,85]. Moreover, protein acetylation has also been proposed to play a significant role in the aging process by improving the function of specific genes, most notably the AMP-activated protein kinase (AMPK) regulatory subunit, which has been linked to increased longevity [86].

### 2.5. Longevity Genes

Scientists propose that the aging process is primarily caused by a genetically programmed continuum of growth and maturation. The maximum lifespan is very species-specific, as humans have a maximum lifespan 30 times longer than mice. In this context, it was discovered that specific genes found in many animals play a role in determining the full lifetime potential [87,88,89]. Indeed, the existence of these genes results in the synthesis of products that are engaged in the control of the species’ life via several mechanisms, including the modulation of stress and resistance, the increase in metabolic capacity, and the silencing of genes that promote aging [90,91,92]. In this respect, numerous investigations have suggested that the overexpression of the SIR2 gene and its homolog increases the lifespan of yeasts and nematodes [93].

Sir2 has been linked to reduced histone acetylation at the amino group of N-terminal lysine residues and global hypoacetylation in yeast [94]. Sirtuins are thought to play an essential role in cell response to several stimuli, including oxidative and genotoxic stress, and are necessary for cell metabolism [95]. Many studies have questioned the direct involvement of sirtuins in extending human lifespan, and their intervention in many human body systems such as the liver and cardiovascular system. It has been suggested that the main activity of sirtuins is the deacetylation of lysine residues [96,97]. Sirtuins cleave nicotinamide adenine dinucleotide (NAD) to nicotinamide. Then, an acetyl/acyl group is transferred from the substrate to the ADP-ribose moiety of NAD, resulting in 2′-O-acetyl-ADP-ribose and a deacetylated substrate [98].

Other researchers suggest that mutations in specific genes, such as the daf-16 gene implicated in various signal transduction pathways, including insulin signaling [93,99,100], may cause greater longevity in mutants [101]. In contrast, genetic research on mammalian longevity has revealed the presence of immunological loci in mice and humans, with these loci having implications for longevity [80]. The role of genetics in longevity was highlighted after scientists discovered that siblings and parents of long-lived people also live longer, and researchers found the presence of multiple genes on chromosome 4 linked to exceptional longevity [102,103,104], most of which are pleiotropy genes. However, since age causes differences in gene expression in muscles and the brain, several studies have shown that caloric restriction prevents age-related gene expression changes in mice. Furthermore, several investigations have focused on the cellular pathway by suggesting that aging is a cellular model, and that an individual capacity is relatively proportional to the functional capacity of the cells [105,106].

Intracellular enzymes such as collagenases, elastases, and tyrosinase are increased by intrinsic aging and photoaging factors, resulting in skin aging [107]. Extrinsic aging is generated by external stimuli, such as chronic exposure to pollutants or UV rays. At the same time, it is believed that internal aging is controlled and established by several hereditary genes [108,109]. Collagen and elastase in the dermis denature as a result of persistent UV exposure and other external factors, leading to wrinkles and the photoaging of the skin; by stimulating intracellular signal transcription pathways such as p38 mitogen-activated protein kinase and c-Jun-N-terminal kinase, this mechanism will induce the creation of MMPs, which can arise from extracellular matrix (ECM) degradation [110,111,112]. Numerous proteins present inside the ECM have been discovered. Elastase is a member of the chymotrypsin family of proteases that is primarily responsible for the degradation of elastin and collagen, essential biomolecules protecting the skin against damages [113]. Several studies have emphasized the critical role of disposing foreign proteins within the ECM during neutrophil phagocytosis and tissue healing in standard settings [107]. Many research works have focused on the direct impact of inhibiting those enzymes. It has been found that inhibiting elastase and MMP-1 enzymes may positively affect skin aging due to their usefulness in avoiding skin sagging and losses of elasticity [107,114]. Furthermore, scientists have investigated the impact of inhibiting tyrosinase, a copper-containing monooxygenase that catalyzes the O-hydroxylation of tyrosine to 3,4-dihydroxyphenylalanine and then to dopaquinone, which is synthesized by epithelial, mucosal, retinal, and ciliary body melanocytes, and which is deeply involved in the protection of the skin from melanogenesis [115,116].

### 2.6. Cellular Senescence

Cellular senescence and the number of divisions are required to determine proliferative lifespan [1,117]. Several studies have shown that a decrease in the rate of cell proliferation corresponds to aging in animals [28,118]. Multiple intrinsic and extrinsic factors, including oxidative stress (OS), DNA damage, oncogene activation, epigenetic stress, and mitotic spindle stress, can cause cellular senescence. When stem cells or progenitor cells are damaged, cellular senescence compromises tissue function and reduces tissue regeneration capacity [119]. In this context, several studies on the senescence of many cells and their relationship with aging, such as glial cells [120], keratinocytes [121], vascular smooth muscle cells [122], lens cells [123], endothelial cells [124], and lymphocytes [125], have been conducted. The results indicate that eliminating senescent cells in adult wild-type mice delays tumor formation; thus, eradicating senescence could help people live longer [126]. In addition, normal cells in life forms have a limited proliferative ability; cells divide less frequently in humans, and their latency before proliferation increases. Despite these investigations, there is no conclusive evidence that senescent cells grow in vivo as people age.

### 2.7. Cell Death

Traditionally, cell death methods have been divided into energy-dependent programmed cell death apoptosis mechanisms and necrotic cell death mechanisms. The body can preserve equilibrium in the apoptosis mechanism by committing active “suicide”. This is a preprogrammed death caused by the progressive activation of a group of cysteine proteases known as caspases. Most cells have caspases in their cytoplasm, but they are generally inactive (procaspase) [127]. Because there is no release beyond the cell, there is no inflammation. This occurs in response to environmental stimuli and is controlled by genes [128,129]. In addition to its essential role in the immune system, where up to 95% of T cells undergo cell death (presumably because they recognize self-antigens) [130], it involves the compaction and segregation of chromatin adjacent to the nuclear membrane and the condensation of the cytoplasm, eventually evolving into nuclear/cellular fragmentation. Meanwhile, apoptosis may play a role in aging and age-related disorders. Apoptosis, followed by the replacement by division of another cell, may occur if cells are unable to repair DNA damage; it is also essential for wound healing, which is often reduced as people age, often in conjunction with local inflammation [131]. The central nervous system shows the most transparent relationship between apoptosis and aging, whereby neuronal apoptosis increases with aging. Similarly, cancer rates rise with age due to decreased apoptotic defenses [132].

## 3. Anti-aging Effects of Vegetables

Numerous investigations have shown that vegetable extracts exhibit significant anti-aging effects. Table 1 lists the different studies pertaining to the anti-aging effects of vegetables that have been investigated.

### 3.1. Allium cepa (Onion)

Kim et al. [134] carried out a study linking the consumption of onions to an anti-aging potential, focusing on different parts (husk, peels, oil, and fermented extract) and different approaches: (i) in vivo using aged rodents and (ii) in vitro using different cell lines and enzymes. The research findings revealed that the extract/fractions of *Allium cepa* peel exhibit antioxidant activity in biological systems, especially skin exposed to UV radiation by scavenging free radicals and other ROS, thus protecting cell membranes against ROS and possibly as anti-aging agents. Similar studies showed that the husk and peels are an excellent source of antioxidants using oxygen-free radical scavenging capacity and ferric reducing antioxidant power assays [133] and DPPH, as well as ROS scavenging activities [134]. Antioxidant activity was also shown in aged male rats (approximately 1.5 years) given the husk extract orally for 188 days, affecting antioxidant system indicators of the liver and brain, but not of the blood and plasma, mainly due to elevations in catalase and superoxide dismutase activity in the liver by 44.4% and 79.1%, respectively, and in the brain by three-fold and 79.1%, respectively.

Similarly, onion peel extract exhibited antibacterial effects against skin resident flora, especially *Staphylococcus aureus* (MIC = 0.06%), and inhibitory effects on tyrosinase (IC_50_ = 9.16 µg/mL) and elastase (IC_50_ = 14.12 µg/mL) [134]. On the other hand, when the onion oil was given to thirty aged rats (1.5–2 years) for four weeks, it reduced the liver and kidney malfunction indicators, total protein and albumin, improved lipid profile, decreased monoamine levels, and increased testosterone level, indicating that onion oil acts as an anti-aging agent [135]. Moreover, the anti-aging potential of the fermented onion extract was investigated [136], and the results reveal that the fermented onion extract effectively suppresses melanin production by inhibiting tyrosinase expression in B16F10 melanoma cells at a concentration of 100 μg/mL. Additionally, the fermented onion extract caused the downregulation of collagenase-1 expression and the upregulation of type I collagen level in UVB-irradiated HaCaT keratinocyte cells. The production of hyaluronic acid was increased to 41.11% (10 μg/mL), 107.78% (100 μg/mL), and 146.67% (200 μg/mL) compared to the UVB-irradiated control. These studies suggest the potential use of onion extract as a potential anti-aging agent, and in preventing or treating melanin pigmentary diseases and UVB-induced wrinkle formation.

### 3.2. Allium sativum (Garlic)

Garlic was researched for its possible anti-aging and rejuvenating properties based on reported therapeutic benefits related to its use as a dietary supplement, such as detoxification, antioxidant, antifungal, antibacterial, and tumor suppressor activities. Different extract types were selected for this purpose, such as aged garlic extract, hydroethanolic and aqueous extracts, and other cell types and animal models. In this line, Moriguchi and coworkers [137] investigated the effect of chronically aged garlic extract against age-related alterations in a new strain of senescence-accelerated mouse (SAM) with age-related brain atrophy (SAMP10). The mice were fed with a diet containing 2% of the aged garlic extract for approximately eight months. The administration of the aged garlic extract prevented the increase in the grading score of SAMPlO and SAMR1, improved the learning and memory deficits of SAMPlO in both the passive and conditioned avoidance tests as well as in the spatial memory test, and prevented the decrease in brain weight and atrophic changes in the frontal brain at 12 months of age. This suggests the beneficial use of aged garlic extract as an anti-aging remedy, especially for age-related cognitive impairment in humans.

Similarly, a group of researchers investigated the protective effects of hydroethanolic garlic extract against photo-damage and cell senescence in UVB-exposed human keratinocytes. The prepared extract (10 mg/mL) demonstrated potent antioxidant activity in the DPPH (87.4 ± 9.0%) and NO (90.4 ± 5.0%) scavenging activity tests in a cell-free system, and attenuated UVB-induced intracellular ROS production by 29.4% at 50 µg/mL. The extract (100 µg/mL) also inhibited the production of UV-induced pro-inflammatory cytokines (IL-6 and IL-1β) and improved the SA-β-gal and SIRT1 activities. The data obtained from this study support the use of garlic as an anti-aging material, especially against photo-aging and cellular senescence [118]. To investigate the garlic’s anti-aging effects on long-term growth characteristics, morphology, and the macromolecular synthesis of human skin fibroblasts, Svendsen et al. [139] used the Hayflick system of cellular aging in culture. This study showed that the addition of garlic extract to standard cell culture medium has youth-preserving, anti-aging, and beneficial effects on human fibroblasts. On the other hand, this treatment prevented the development of malignant cells, which could not grow longer in the presence of garlic.

### 3.3. Allium tuberosum (Chinese Chives, Buchu)

Lee and coworkers [140] investigated the protective effect of *Allium tuberosum* on the skin of ICR mice fed with diets containing 2% or 5% *Allium tuberosum* (Chinese chives, buchu) for 12 months. In this study, researchers assessed the antioxidant enzyme activity, total glutathione concentrations, and insoluble collagen content in the skin with or without UVB irradiation. Compared to the control group, dietary buchu reduced the thiobarbituric acid reactive substances (TBARS) and protein carbonyl levels in the skin. Those given the 5% buchu diet showed levels below those fed a 2% buchu diet. In the group fed with the control diet, antioxidant enzyme activities and total glutathione concentrations in ICR mice decreased with age. In contrast, enzyme activities and glutathione concentrations in the buchu diet groups remained normal throughout the study. In the skins of buchu-fed mice, SOD, GPx, CAT activity, and total glutathione contents increased over time. Dietary buchu effectively reduced the lipid peroxidation and protein oxidation induced by UVB irradiation in ICR mouse skin homogenates. Compared to the control group, the buchu diet reduced the synthesis of non-soluble collagen in mouse skin. These findings imply that buchu’s antioxidant components and sulfur compounds may protect against the oxidative stress caused by aging and UV irradiation.

### 3.4. Dioscorea aimadoim (Yam)

Yam (*Dioscorea aimadoimo*) has been recognized as a nutritious food due to its biological activities, including anti-obesity, anti-constipation, anti-mutagenic, and hypoglycemic and cholesterol-lowering properties. Researchers have investigated the effects of the extraction conditions on its antioxidant and moisturizing capacity, collagenase activity, proliferation, and migration to assess its anti-aging potential [141]. The high- and low-temperature ethanol (400 mg/mL) extract of *D. aimadoimo* (HAD and LDA) showed 70.6% and 40% electron-donating capacities, respectively. Moreover, the SOD-like activities of LDA and HDA were 23% and 34%, respectively. LDA, compared to HDA, considerably lowered collagenase activity in a dose-dependent manner. The water content of LDA- and HDA-treated skin was 45.63% and 38.65% higher than that of the placebo cream, respectively. The administration of LDA and HDA at a 200 mg/mL dose increased cell proliferation to 109.7% and 114%, respectively, compared to the control. Furthermore, the ethanol extract of *D. aimadoimo* could be a highly effective anti-aging and skin moisturizing cosmetic component.

### 3.5. Dioscorea opposite (Yam)

Wang et al. [142] used hot water extraction and ethanol precipitation to extract Chinese yam polysaccharides (CYP) from the tuber of *D. opposite*. The main objective was to evaluate the anti-aging activities of CYP in aging mice induced by d-galactose. The results reveal that CYP could improve the mice’s learning abilities and help them recover from spatial memory deficits. Furthermore, in the brain, liver, and renal tissues of mice, the CYP efficiently inhibited the generation of malonaldehyde (MDA) and increased the activities of SOD, CAT, and GPx. In addition, the histological study revealed that CYP could significantly reduce d-galactose-induced damage in the mentioned tissues and could, according to gene expression studies, upregulate the expression of the anti-aging klotho gene in the brain and kidneys. Thus, the anti-aging effect of Chinese yam was achieved by repairing organ function and enhancing the expression of the klotho gene in animals.

### 3.6. Asparagus cochinchinensis (Chinese Asparagus)

*Asparagus cochinchinensis*, commonly known as Chinese asparagus, is a traditional Chinese medicine used to treat various diseases, including age-related. Therefore, researchers have studied the anti-aging potential of extracts from the root, stem, and shoot of this plant using multiple animals and cellular models [143,144]. In this respect, Xiong et al. [143] investigated the effects of root and stem extracts of *A. cochinchinensis* on biochemical indicators related to aging in mouse brains and livers. For this purpose, SOD activity, MDA content, and total protein content in the mouse brain, liver, and plasma were compared before and after the extract treatment. The results demonstrated that the polysaccharides and aqueous extracts of the roots effectively improved the spleen index and SOD activity, while lowering MDA levels and slowing the aging process. On the other hand, the stem extracts dramatically decreased SOD activity and increased MDA accumulation in the brains and livers of mice, suggesting that the stem extracts may not be suitable for treating aging-related disorders.

In another study, Lei and colleagues [144] investigated the antioxidant potential of the *A. cochinchinensis* shoot as a potential mechanism explaining its anti-aging proprieties in the d-galactose-induced mouse aging model. This investigation showed that the aqueous extract of *A. cochinchinensis* exhibits good antioxidant activities in the DPPH and ABTS tests, and significantly increased NOS, CAT, and SOD activities and NO content, while substantially lowering MDA content in the aging model mice. Furthermore, in the treated group, the microstructure of mice viscera was noticeably improved, and the expressions of NOS, SOD, and GPx were enhanced. In vivo and in vitro results of this study support the potential of *A. cochinchinensis* shoot aqueous extract to reduce radicals in the body and prevent aging.

Similarly, Lei et al. [145] investigated the antioxidant potential of *A. cochinchinensis* root extract, which could explain the anti-aging proprieties of the extract. This extract exhibited similar free radical scavenging activities to vitamin C. Additionally, the aqueous extract boosted white blood cell count and SOD, CAT, and NOS activities in aged mice. Furthermore, the aqueous extract raised NO concentration while lowering MDA content, suggesting that the root extract may help to prevent aging by scavenging free radicals.

### 3.7. Asparagus officinalis (Asparagus)

Different preparations (tip and spear and steam extract) of asparagus (*Asparagus officinalis* L.) were studied by Sriyab et al. [146] and Shirato et al. [147] for nutricosmetic effects, especially the anti-aging potential. Sriyab et al. [146] aimed to evaluate the nutricosmetic effects of *A. officinalis* extracts. The tips and spears of *A. officinalis* were extracted with 95% ethanol. The most effective extract was derived from the spears, which inhibited MMP-1, elastase, and hyaluronidase at 83%, 70.4%, and 75.2%, respectively. Surprisingly, the *A. officinalis* spear extract was more effective in inhibiting MMP-1 than the well-known natural MMP-1 inhibitors oleanolic acid and epigallocatechin gallate at the same dose. These findings indicate that *A. officinalis* extract is a promising natural anti-wrinkle agent.

Similarly, Shirato et al. [147] investigated the effects of *A. officinalis* stem extracts on HSP70 expression levels in UV-B-irradiated normal human dermal fibroblasts to determine their anti-photoaging properties (NHDFs). After 1–6 h of culture, UV-B-irradiated NHDFs showed lower HSP70 mRNA levels, which were restored after 24 h. HSP70 mRNA levels in the NHDFs increased after 24 h of treatment with the extract alone, but this was not reflected in protein levels. On the other hand, pretreatment with the extract prevented the UV-B-induced decline in HSP70 expression at mRNA and protein levels. These findings imply that the extract may retain the levels of HSP70 in UV-B-irradiated NHDFs, thus demonstrating anti-photoaging properties by inhibiting the downregulation of HSP70 expression in UV-irradiated dermal fibroblasts.

### 3.8. Amaranthus tricolor (Red Spinach)

Wrinkling and a lack of suppleness in the skin are evident signs of changes. Recognizing that skin aging is a complex natural phenomenon that involves the skin’s structural integrity and physiological function deteriorating over time, Amelia et al. [148] tried to see how successful red spinach extract was in increasing collagen, elasticity, hydration, sebum, and pigment in male rats. Skin moisture levels before and after treatment with the red spinach extract ointment showed a significant difference, with the most significant change occurring at 10% concentration (64.84%). The skin collagen levels changed significantly before and after treatment, with the most significant change reported at 10% concentration, or 56.25%. In addition, significant increases in skin elasticity were recorded before and after treatment, with the biggest change observed at a 10% concentration, namely, 46.30%. Skin pigmentation levels changed significantly before and after treatment, with the most significant change occurring at the 10% concentration (35.97%). The maximum concentration of 10% also resulted in the most significant percentage reduction in sebum level, which was 40%. Likewise, the red spinach leaf extract ointment exhibited effective anti-aging action.

### 3.9. Cynara scolymus (Artichoke)

*Cynara scolymus* artichoke was chosen in different studies to investigate its use as a potential anti-aging remedy. In this context, Marques et al. [150] attempted to create a topical formulation and investigated its activity as an antioxidant and sun protector. The prepared artichoke extract was rich in polyphenols and was incorporated into two topical formulations: emulsion and hydrogel. The preparation’s quality, safety, and efficacy were confirmed by various microbiological controls, cytotoxicity assays, and ROS scavenging activity tests in HaCaT cells. In the in vivo tests, Human Repeat Insult Patch Testing indicated an excellent antioxidant and photoprotective activity. The formulation prepared in this study was safe and effective for use in dermal application and as a promising new anti-aging cosmetic ingredient. Similarly, Song and colleagues [149] assessed the antioxidant ability of artichoke leaf extract in d-galactose-induced aging rats. The results obtained indicated that the activity of SOD in the brain and liver, GPx in the brain, and CAT in the liver increased significantly compared to the aging model group. In contrast, the MDA content in the serum, and LF in the brain and liver, decreased significantly. These results support the use of artichoke preparation in oxidative stress-related disorders.

In addition, Sukoyan et al. [151] evaluated the antioxidant potential of standardized artichoke extracts (2%) as a protective strategy against skin age-associated oxidative damage caused by d-galactose in rats. These researchers discovered that a low dose of artichoke extracts (2%), by intradermal microinjection, triggers the enzymatic link in the innate antioxidant defense system in a skin aging model, thus suggesting its use in cosmetics as anti-aging mesotherapy. Besides this, Sukoyan and coworkers [152], using the same skin aging model, studied the effect of artichoke extract in reverse disturbances of collagen metabolism and inflammation. Consequently, this treatment restored relative skin weight, increased neutral salt and acid solubility, decreased the collagen fraction of pepsin solubility, restored the hexosamine/collagen (hydroxyproline) ratio, and decreased NF-κB activity. In this model of d-galactose-induced skin aging, long-term therapy with artichoke extracts enhanced collagen metabolism and slowed inflammation progression.

### 3.10. Taraxacum officinalis (Dandelion)

Many older people have andropause symptoms, such as a lack of physical and mental activity. In this context, research findings [153] have indicated that *Taraxacum officinalis* (Dandelion) extract activates the ERK and Akt pathways, which protect TM3 cells against serum restriction and oxidative damage. In rats, testosterone levels and spermatogenesis activation were dramatically increased, and physical movements were also significantly enhanced. According to a clinical study, the daily consumption of 400 mg of the extract increased the quality of life of aged male rats. These findings suggest that dandelion has the potential to be a safe and effective natural drug for alleviating or treating andropause symptoms.

### 3.11. Brassica oleracea L. var. capitata F. rubra (Red Cabbage)

Exposure to UV-B radiation causes photoaging in the dermal layer of the skin. Krishnan et al. [154] evaluated the histological characteristics of the dermis layer in male Wistar mice exposed to UV-B after applying a cream prepared from the ethanolic extract of red cabbage. The results obtained indicated no statistically significant difference in the thickness of the dermal layer among groups, thus suggesting that red cabbage ethanol extract does not show any significant effect on the thickness of the dermis in male Wistar mice.

### 3.12. Brassica oleracea L. var. italica Plenck (Broccoli)

UVB irradiation induces MMP production by activating the cellular signaling transduction pathways responsible for collagen degradation. In this regard, researchers have investigated the effects of broccoli flower extract on photoaging inhibition and MMP-1 expression in human skin fibroblasts [134]. Pretreatment with the extract reduced MMP-1 expression at both the mRNA and protein levels. In addition, the extract caused significant changes in MMP-1, mRNA, and MMP-1 protein expression. We can thus say that broccoli extract is a potential anti-photoaging agent.

### 3.13. Raphanus sativus (Radish)

Radish skin and greens (mucheong) are the plant’s edible parts. However, due to their harsh and abrasive texture, they are removed before consumption and used as a by-product or animal feed material. In this respect, Kim [156] examined how supercritical heat-treated radish extract affect anti-aging wrinkles in UV-induced Hos: HRM-2 wrinkled mouse animal models. The results showed that the supercritical heat-treated radish extract, when applied to the skin taken orally, helps in reducing wrinkles. As a result, radish extract may be employed as a component in health-related products. Even after long-term consumption, this vegetable can prevent wrinkle development and enhance skin suppleness when consumed regularly, with no known adverse effects.

### 3.14. Cucumis sativus (Cucumber)

Research by Nema and collaborators was conducted [157] to determine if the lyophilized juice of *C. sativus* exhibits antioxidant, anti-hyaluronidase, and anti-elastase activity. The results obtained indicate that the extract exhibits good antioxidant activity, and shows strong anti-hyaluronidase and anti-elastase activities. These researchers concluded that *C. sativus* could be used as an anti-wrinkle product in cosmetics due to the extract’s high ascorbic acid content.

### 3.15. Cucurbita moschata (pumpkin)

Pumpkin seed (*Cucurbita moschata*), which contains tocopherol as an antioxidant, is a good candidate for development as an anti-aging drug. In this respect, Muntafiah and colleagues [158] looked into the anti-aging effects of pumpkin seed extract on doxorubicin-induced normal NIH-3T3 fibroblasts. The results indicate that the extract is not cytotoxic for NIH 3T3, and at 100, 200, 400, and 800 μg/mL, it reduced cell senescence percentage by 2.77, 4.5, 6, and 18 times, respectively. Meanwhile, an in silico investigation revealed that tocopherol interacts with CYP 3A4 more strongly than doxorubicin. Pumpkin seed extract exerts its anti-aging effect by decreasing SA-gal activity. Based on molecular docking, this anti-aging function could be related to tocopherol’s interaction with CYP 3A4.

### 3.16. Glycine max (Soybean)

Soybean (*Glycine max*) is one of the most broadly exploited natural products in discovering new bioactive anti-aging agents. Different extracts have been prepared, such as protein hydrolysate, alcoholic extract, soy pulp, monascus-fermented extract, fermented soybean milk, and soybean peptides. In the study by Amakye et al. [159], the protein hydrolysate had the potential to reverse learning associated with d-galactose-induced aging, memory impairment, and oxidative stress. In contrast, the alcoholic extract [139] that was added to a cosmetic emulsion containing 4% of the extract, which was proven to have beneficial effects on skin elasticity and moisture contents, reduced skin scaliness, skin wrinkles, skin smoothness, and skin roughness after 12 weeks of application. Similarly, Li et al. [163] selected soymilk fermented by *Lactobacillus plantarum* to investigate the antioxidant and anti-aging effects. The fermented milk demonstrated a better antioxidant activity than non-fermented, and the histopathological observation showed that the extract could protect the skin, spleen, and liver, and reduce oxidative damage and inflammation. The extract effectively upregulated *GSH*, *CAT*, *SOD*, and *GPx* levels, decreased *MDA* content in the liver, brain, and serum, and promoted relative gene expression levels encoding for cuprozinc superoxide dismutase (*Cu/Zn-SOD*, SOD1), manganese superoxide dismutase (*Mn-SOD*, SOD2), *CAT*, GSH, and *GPx* in the liver, spleen, and skin. On the other hand, Wu et al. [164] confirmed the antioxidant activity of black soybean peptide in aging mice caused by d-galactose. In contrast, the study by Corpuz et al. [161] on the oral administration of okara soybean by-products recorded the attenuation of cognitive impairment in a mouse model of accelerated aging. Lastly, Jin and Pyo [162] also confirmed the effect of monascus-fermented soybean extracts on antioxidant and skin aging-related enzyme inhibitory activities. These studies support the use of soybeans as natural nutricosmetic and anti-aging products.

### 3.17. Vigna angularis (Red Bean)

Prasetyo and coworkers [165] conducted a study to investigate the physical proprieties and anti-aging effects of red bean extract as a peel-off gel mask. The entire formula was homogeneous, with a pH of about 6, a peeling time of less than 20 min, minimal irritation, and a shelf life of 12 weeks at room temperature. The moisture level, pore size, evenness, and count of black spots all improved after treatment with the formula (5% red bean extract being the best). These results suggest that a red bean ethanol extract may be made as a peel-off gel mask with anti-aging properties.

### 3.18. Phaseolus vulgaris (Black Bean)

Hernandez and coworkers [166] evaluated the phenolic extracts from black bean (*Phaseolus vulgaris* L.) for their possible antioxidant and anti-aging potential. The best extraction conditions for obtaining phenolic compounds and anthocyanins were water–ethanol (50%) as co-solvents; this afforded 66.60 ± 7.4 mg GAE/g coat (total phenolic compounds) and 7.3 ± 0.6 mg C3GE/g coat (total anthocyanin content). The extract exhibited an excellent DPPH and ABTS antioxidant activity. Finally, the IC_50_ values for the enzymatic inhibition assays of tyrosinase, collagenase, and elastase were 10.44 ± 1.32, 8.33 ± 0.65, and 0.11 ± 0.02 mg GAE/g coat, respectively. The black bean extract exhibited high antioxidant capacity and inhibitory potential against tyrosinase and metalloproteinases, such as collagenase and elastase. Furthermore, black bean phenolic extracts could be used in cosmeceutical products to prevent oxidative stress and aging.

### 3.19. Vigna angularis

Based on *Vigna angularis*’ (Azuki bean) long history of use both as a food and as a traditional medicine, Hwang et al. [167] conducted work to validate its functional applicability in dietetics and cosmetics for skin protection. The antiphotoaging effects of a hot-water extract from Azuki beans were studied. This study showed that MMP-1 synthesis was considerably reduced in UVB-exposed normal human dermal fibroblasts treated with the 90% extract. In UVB-irradiated hairless mice, both topical and oral administration of the extract increased elastin, procollagen type I, and TGF-b1 expression by 118%, 156%, and 136%, respectively, and decreased MMP-1 synthesis. Additionally, the extract reduced wrinkle development and skin thickness. These findings imply that *V. angularis* extract may be helpful in reducing UVB-accelerated skin photoaging.

### 3.20. Abelmoschus esculentus (Okra)

Senescence has recently been linked to an excess of ROS and the over-activation of the senescence-associated galactosidase (SA-gal) before synaptic plasticity loss. Within this context, Jumnongprakhon et al. [147] examined the activity of the *Abelmoschus esculentus* (L.) (Okra) ethanolic extract against neuronal aging. In their study, SK-N-SH cells were pretreated with H_2_O_2_ for 4 h before being treated with different extract concentrations. The results show that the extract significantly increases cell viability, while reducing ROS concentration and SA-gal positive cells. Additionally, the AChE activity was reduced, and the overall synaptic plasticity was improved. This study suggests that the Okra extract may reduce the negative responses in aged neurons.

### 3.21. Rheum rhabarbarum (Rhubarb)

The effect of *Rheum rhabarbarum* (Rhubarb) on UVB-induced HaCaT human skin aging was investigated by Ying et al. [169]. Compared to the UVB group, rhubarb significantly reduced the expressions of P38, TNF-α and IL-6. Based on these results, rhubarb phenol can greatly slow the aging of HaCaT cells, and its mechanism may be related to blocking P38 signaling pathways and inflammatory cytokine release.

### 3.22. Rumex crispus (Curly Dock)

Uzun and Demirezer [170] suggested that the inhibition of MMP enzymes, and higher sun protector and antioxidant capacity, should be a part of the treatment strategy to prevent skin aging. To this end, they investigated the anti-aging potential of *Rumex crispus* L. (curly dock) extracts by determining the MMP-1, MMP-8, and MMP-13 inhibitory effects, UV absorption, and antioxidant capacities. The data obtained from this study indicate that the hydroethanolic extract exhibits the highest inhibitory effect against all MMP enzymes, high UV protection, and a total antioxidant capacity between 49.4% and 86.4%. Likewise, *Rumex crispus* L. was beneficial in this research, providing a valuable and effective source for anti-wrinkle, antioxidant, and sunscreen cosmetics.

### 3.23. Daucus carota (Carrot)

As the prevention of neurodegenerative illnesses is aided by brain protection against accelerated aging, Mohamed et al. [171] evaluated the potential use of *Daucus carota* (carrot) seeds to prevent brain aging induced by d-galactose in rats. The results show that the tested extracts suppressed both the reduction in CAT and the elevation in MDA, in either brain or plasma, and the increase in plasma TNF-α and BChE, as well as liver and kidney parameters. Carrot seeds can serve as potential protective agents against the accelerated aging parameters, which may be due to their antioxidant and anti-inflammatory activities.

## 4. Anti-Aging Natural Compounds from Vegetables

Recent investigations have shown that bioactive compounds from different medicinal and nutritional species exhibit remarkable anti-aging effects. Other mechanisms are involved in these effects, including their capacity to upregulate senescence, induce apoptosis, and increase cell longevity. Investigations on the anti-aging properties of natural compounds isolated from vegetables are outlined in Table 2.

### 4.1. Allicin (Allium sativum)

**Figure 2:**
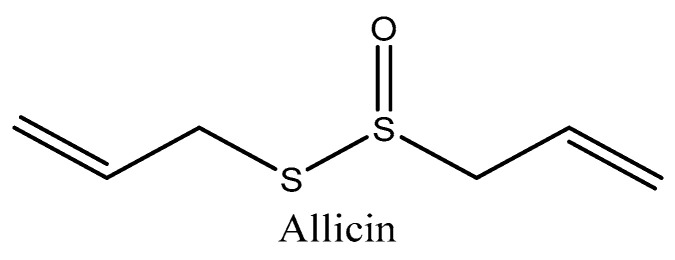
Chemical structure of allicin.

Pangastuti and coworkers [172] used molecular docking against leukocyte elastase as a protein target to examine the beneficial components of *Allium sativum*, specifically allicin (Figure 2). Leukocyte elastase is a protein responsible for elastin degradation, which subsequently inhibits OS and the apoptosis cycle and allows this protein to be involved in aging. For an effective therapy of aging, the inhibition of leukocyte elastase activity could be proposed as a strategy in the future. Compared to other reference compounds of this study, allicin has the highest binding affinity (−8.7 kcal/mol) to leukocyte elastase, indicating that it is the most promising candidate for premature aging therapy. Allicin inhibited leukocyte elastase, with hydrogen bonding and hydrophobic interactions enhancing binding stability. This was explained by the interaction of leukocyte elastase with four residues, some of which are part of its primary structure, namely, Val 216, Phe 215, ser 214, and Phe 192. The residues Val 216 and Phe 192 were the active sites on this protein. In addition, due to Lipinski’s rule, allicin is a promising anti-aging drug. 

#### Caffeic Acid, S-Allyl Cysteine, and Uracil (*Allium sativum*)

**Figure 3:**
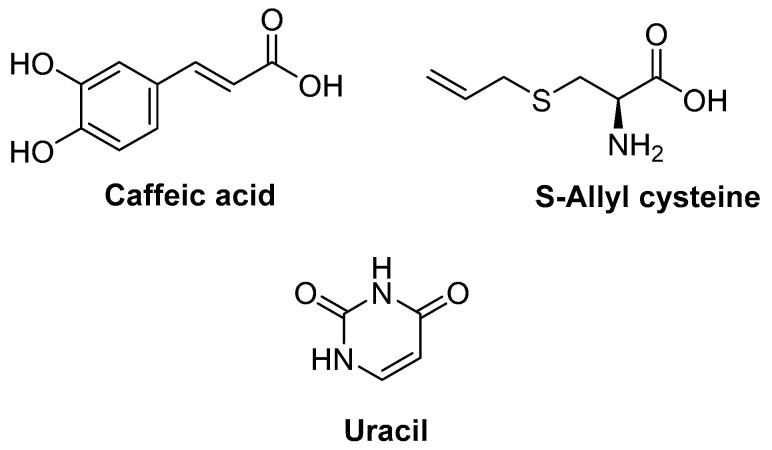
Chemical structures of caffeic acid, S-allylcysteine, and S-ally-uracil.

UV irradiation, stress, and tobacco contribute to skin aging, a multisystem degenerative process. Wrinkle production is also a prominent aspect of photoaging, and it is linked to oxidative stress and the inflammatory response. Kim and colleagues [173] examined whether caffeic acid, S-allyl cysteine, and uracil (Figure 3), all of which were extracted from garlic, could control UVB-induced wrinkle development and affect the expression of MMP and NF-κB signaling. The results revealed that the three compounds strongly inhibited type I procollagen degradation and MMP expression in vivo, and reduced histological collagen fiber disorder and oxidative stress. Caffeic acid and S-allyl cysteine also reduced OS and inflammation by modulating NF-κB and AP-1 activities. At the same time, uracil had an indirect antioxidant effect by suppressing COX-2 and iNOS expression levels, and downregulating transcriptional factors. These findings show that the anti-wrinkle properties of caffeic acid, S-allyl cysteine, and uracil are due to their antioxidant and anti-inflammatory properties.

### 4.2. Glucosinolate (Glucoraphanin and Sulforaphane) and Phenolic (Kaempferol and Quercetin) from Broccoli (Brassica oleracea)

**Figure 4:**
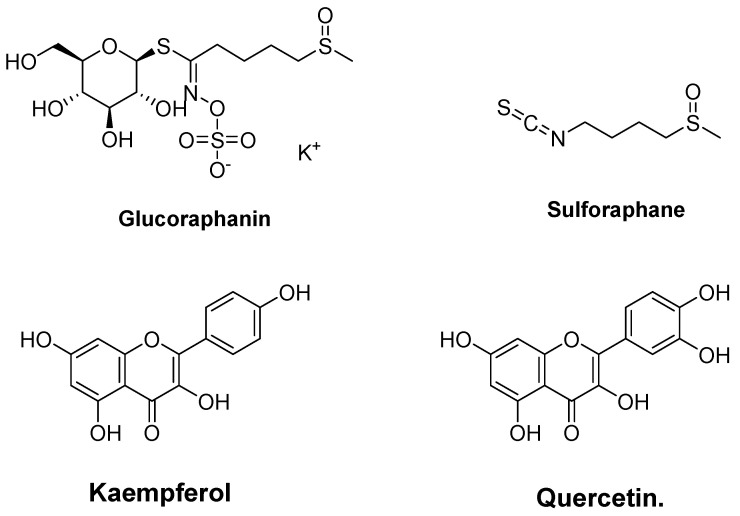
Chemical structures of glucoraphanin, sulforaphane, kaempferol, and quercetin.

Because aging is caused by an imbalance between antioxidants and ROS, Hikmawati et al. [174] investigated whether the Keap1 receptor could be inhibited by some of broccoli’s (*Brassica oleracea*) bioactive compounds (Glucosinolate (glucoraphanin and sulforaphane) and phenolics (kaempferol and quercetin)) (Figure 4). The results show that the binding energy of quercetin with Keap1 was −268.72 kcal/mol, and glucoraphanin with Keap1 was −318.01 kcal/mol. In addition, both compounds could inhibit the Keap1–Nrf2 interaction. Consequently, Nrf2 could transcribe antioxidant genes. The interaction between Keap1 and quercetin may be related to certain ROS-lowering actions, such as enhanced HMOXI expression. This study indicates that quercetin has greater potential in drug development as a peroxidase inhibitor.

### 4.3. Water-Soluble Protein (Vicia faba)

Okada and Okada [176] isolated a free-radical scavenger “water-soluble protein (WSP)” from beans. The cytosolic SOD activity decreased, whereas the activity of CAT and GSH increased. WSP treatment was overall related to the delay of cellular aging-dependent degeneration.

### 4.4. Arctigenin, Matairesinol, Arctiin, (iso)lappaol A, lappaol C, and lappaol F. (Arctium lappa)

**Figure 5:**
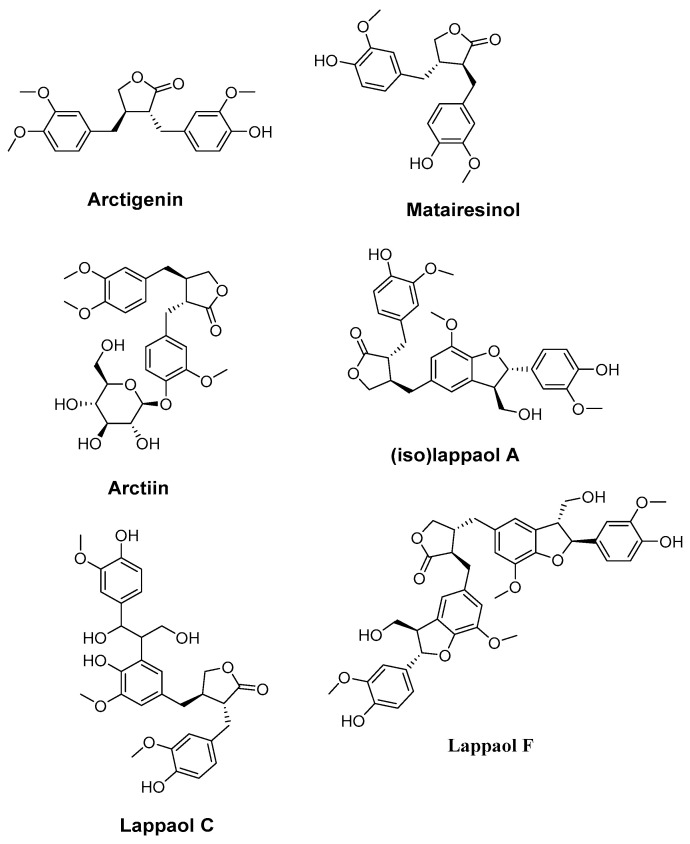
Chemical structures of arctigenin, matairesinol, arctiin, (iso)lappaol A, lappaol C, and lappaol F.

Su and Wink [177] extracted various bioactive compounds (Arctigenin, matairesinol, arctiin, (iso)lappaol A, lappaol C, and lappaol F.) (Figure 5) from *Arctium lappa,* and investigated the anti-aging potential of this plant. *Caenorhabditis elegans* was used as a model to study the antioxidant and anti-aging effects of the separated lignans. All lignans considerably increased the average lifespan of *C. elegans*. The most significant impact was shown with matairesinol, which increased worm lifespan by 25%. Additionally, these researchers found that five lignans are effective free radical scavengers (in vitro and in vivo), and that all lignans may help *C. elegans* survive oxidative stress. Furthermore, lignans may cause the transcription factor DAF-16 to translocate to the nucleus and upregulate its expression, indicating that a DAF-16-mediated signaling pathway may be involved in the reported longevity-promoting effect of lignans. The expression of jnk-1 was upregulated by all lignans, suggesting that lignans may improve the lifespan and stress tolerance of *C. elegans* through a JNK-1-DAF-16 cascade. Thus, the anti-aging properties of lignans could be used to produce anti-aging drugs.

### 4.5. Dioscin, Allantoin, and Diosgenin (Dioscoreae Rhizoma)

**Figure 6:**
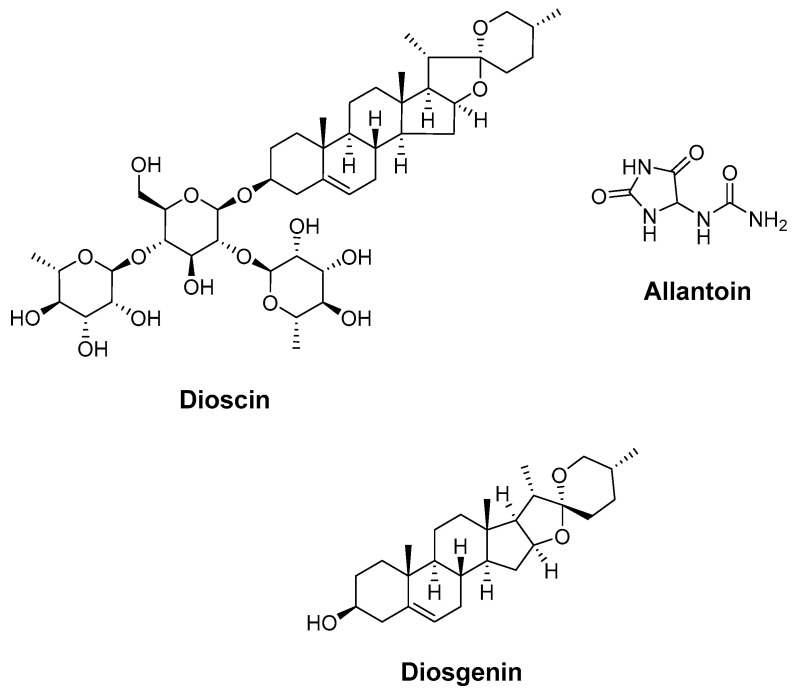
Chemical structures of dioscin, allantoin, and diosgenin.

Chen et al. [178] studied the anti-aging effects of some of the active compounds of Dioscoreae *Rhizoma* (dioscin, allantoin, and diosgenin) (Figure 6). Their findings indicate that these bioactive molecules regulate the expression of target proteins through enriched signaling pathways (covering PI3k-Akt and Rap1 signaling pathways), inhibit tumor proliferation and metastasis, regulate metabolism, and promote nerve repair by regulating the expression of targets (MAPK3, HADC3, HADC1, RXRA, STAT3, etc.) via multiple pathways: the Notch signaling pathway, EGFR tyrosine kinase inhibitor resistance, and the PI3K-Akt signaling pathway.

### 4.6. Glycoprotein (Daucus carota)

**Figure 7:**
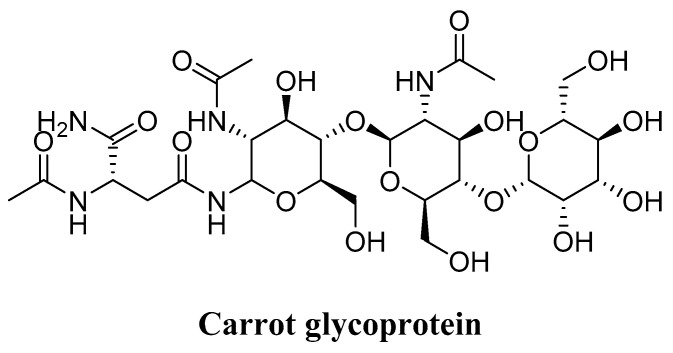
Chemical structure of carrot glycoprotein.

Lee and coworkers [175] investigated the anti-aging activities of the glycoprotein structure shown below (Figure 7) [175]. The results indicate that the glycoprotein exhibits good antioxidant activity and higher lipid peroxidation than BHA and vitamin E. In addition, the glycoprotein was safe, with no toxicity against human dermal fibroblasts. Moreover, it showed efficacy by promoting collagen type 1 generation and reducing MMP-1 activity and ROS clearance. Thus, the carrot glycoprotein could be a potent antioxidant and anti-aging product.

### 4.7. Trp-Pro-Lys (WPK) and Ala-Tyr-Leu-His (AYLH) Glycine max (Soybean)

**Figure 8:**
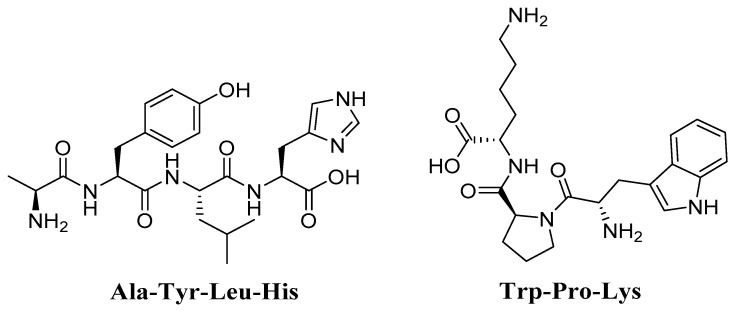
Chemical structures of Trp-Pro-Lys (WPK) and Ala-Tyr-Leu-His. (AYLH).

A group of researchers succeeded in isolating two peptides from *Glycine max* (Soybean), (Trp-Pro-Lys (WPK) and Ala-Tyr-Leu-His (AYLH) (Figure 8) [159], and evaluated their anti-aging potential. The results reveal that peptides strongly attenuate H_2_O_2_-induced oxidative damage in PC12 cells, suggesting that peptides could be potentially effective antioxidant agents in functional foods or nutraceuticals for aging-related learning and memory impairments, and oxidative stress.

## 5. Conclusions

In summary, this review focused on the mechanisms behind aging, and factors that can contribute to triggering premature aging, in particular epigenetic and pathological risk factors. In addition, premature aging can lead to specific pathologies, including neurodegenerative and cardiovascular diseases. Additionally, this review highlighted the effects of certain vegetables as anti-aging agents. It is essential to point out that accumulated stress can lead to very rapid cellular aging during this post-pandemic period. Moreover, the vegetable-derived substances mentioned in this work are highly recommended as food compliments. Indeed, it has been shown that the anti-aging effect of vegetables could essentially be due to the presence of several bioactive molecules belonging to the secondary and primary metabolites. These bioactive molecules exert anti-aging effects via different mechanisms, such as the inhibition of cell senescence, anti-apoptotic effects, and actions on the cycle and cell memory. However, the pharmacokinetics of these bioactive molecules remains incompletely elucidated, and consequently, the selectivity is still poorly understood.

## Figures and Tables

**Table 1:** Anti-aging properties of vegetables.

Vegetables (Common Names)	Extract Types	Models Used	Methods	Key Results	References
**Amaryllideae**					
*Allium cepa* L. (Onion)	Ethanolic onion husk extract	Aged male Wistar albino rats (17 months)	Ferric reducing antioxidant power (FRAP) assay Quantitative determination of reduced glutathione (GSH) Estimation of catalase (CAT) and superoxide dismutase (SOD) activity	Affected the antioxidant system of the liver and brain (for 188 days of treatment) without affecting blood and plasma	[133]
*Allium cepa* L. (Onion)	Ethyl acetate extract from onion peel	Activities investigated in vitro	Antibacterial effect against skin resident flora *Staphylococcus aureus*, *Propionibacterium acnes*, *Pityrosporum ovale*, and *Escherichia coli*1,1-Diphenyl-2-picrylhydrazyl radical, (DPPH) assay Tyrosinase and elastase inhibitory activity	Induced MIC values of 0.06% on skin resident flora Induced excellent DPPH radical scavenging activity (FSC_50_ = 5.05 µg/mL) Induced significant ROS scavenging activity (OSC_50_ = 0.05 µg/mL) Inhibited tyrosinase activity (IC_50_ = 9.16 µg/mL)	[134]
*Allium cepa* L. (Onion)	Onion oil	Male aged rats (1.5–2 years old)	A treatment period of 4 weeks Biochemical measurements	Reduced the elevated levels of all liver function markersReduced total protein and albumin levels Decreased the concentration of urea and creatinine Reduced cholesterol, triglyceride, and LDL levels Increased monoamine levels in aged rats Increased testosterone levels in aged rats	[135]
*Allium cepa* L. (Onion)	Fermented onions extract	B16F10 melanoma cells and HaCaT keratinocyte cells	Cytotoxicity test Melanin content assay Western blot analysis Hyaluronic acid production assay Phytochemical analysis by HPLC-MS	Inhibited melanin formation, at a dose of 100 μg/mL Downregulated collagenase-1 expression and upregulated type I collagen level in UVB-irradiated HaCaT keratinocyte cells Enhanced hyaluronic acid synthesis	[136]
*Allium sativum* L. (Garlic)	Aged garlic extract (AGE)	The SAMPIONS and SAMRl/HS substrains of SAM mice model (a strain of senescence-accelerated mouse (SAM) characterized by age-related brain atrophy)	Evaluation of senescence degree Motor activity Spatial memory test Macroscopic brain morphometry	Prevented the increase in the grading score of SAMPlO and SAMR1 Improved learning and memory deficits of SAMPlO Prevented decreased brain weight and atrophic frontal brain changes at 12 months of age	[137]
*Allium sativum* L. (Garlic)	Hydroethanolic extract	Immortalized human keratinocyte cell line	Antioxidant activity Cell culture and UV irradiation Quantitative real-time RT-PCR MMP-1 production Cytokine determinations	Induced strong DPPH radical scavenging activity (IC_50_ = 2.50 mg/mL) Induced strong NO scavenging activity (IC_50_ = 4.38 mg/mL) Attenuated UVB-induced intracellular ROS production Reduced MMP-1 level and MMP-1 mRNA and protein expressions Inhibited the production of UV-induced pro-inflammatory cytokines (IL-6 and IL-1β) Enhanced SA-β-gal and SIRT1 activities in UV-irradiated HaCaT human keratinocytes Inhibited photoaging due to increased cellular senescence in HaCaT cells	[138]
*Allium sativum* L. (Garlic)	Garlic aqueous extract	Human skin fibroblasts	Cell culture and lifespan estimation Macromolecular synthesis	Sustained serial subcultures for more than 55 population doublings in 475 days Prevented the development of malignant cells	[139]
*Allium tuberosum* Rottler ex Spreng. (Chinese chives, Buchu)	Buchu powder	Male ICR mice	12-month diets containing 2% or 5% buchu Measurement of skin lipid peroxides, protein oxidation, antioxidant enzyme activities, and GSH levels Measurement of insoluble collagen in the skin Ultraviolet irradiation of skin homogenates	Reduced protein carbonyl levels in the skin Maintained enzyme activities and GSH concentrations at youthful levels Increased, over time, the activity of SOD, GPx, and CAT, as well as the total GSH contents Reduced lipid peroxidation and protein oxidation in ICR mouse skin homogenates Reduced the synthesis of insoluble collagen in the skin of mice	[140]
**Dioscoreaceae**					
*Dioscorea aimadoimo* (Yam)	Ethanolic extract	Human dermal fibroblast neonatal (HDFn)	DPPH assay SOD activity Collagenase inhibition Measurement of skin moisturizing effect Cell proliferation rate measurement Effect on cell migration and fibroblast proliferation	Reduced the activity of collagenase Increased skin water content (38–45%) Raised cell proliferation to 114% Induced cell migration in HDFn	[141]
*Dioscorea opposite* Thunb. (Yam)	Yam polysaccharides	Mice	Polysaccharide characterization and determination of their content FT-IR spectroscopy Visceral index and biochemical assay RNA isolation RT-PCR Western blot analysis	Improved the learning abilities of mice and helped them recover from spatial memory deficits Inhibited malondialdehyde generation and increased SOD, CAT, and GPx activities in multiple organs Reduced damage caused by d-galactose in different tissues Upregulated anti-aging klotho gene expression in brain and kidney Restored organ function and improved klotho gene expression	[142]
**Asparagaceae**					
*Asparagus cochinchinensis* (Lour.) Merr. (Chinese asparagus)	Polysaccharides roots and stems aqueous extracts	Mice	SOD activity Malonaldehyde (MDA) and total protein content in the brain, liver, and plasma	Improved spleen index and SOD activity by lowering MDA levels and slowing the aging process Reduced SOD activity and increased MDA accumulation in mouse brain and liver	[143]
*Asparagus cochinchinensis* (Lour.) Merr. (Chinese asparagus)	Shoot aqueous extract	Kun Ming mice	Mouse treated with d-galactose, vitamin C, and extract Measurement of blood cells, nitric oxide synthase (NOS), CAT, SOD, and nitric oxide (NO) activities, and MDA concentration Histopathology	Exhibited good DPPH and ABTS radical scavenging capabilities Increased NOS, CAT, and SOD activities and NO content Reduced MDA content Improved microstructure of mouse viscera Increased expressions of NOS, SOD, and GPX	[144]
*Asparagus cochinchinensis* (Lour.) Merr. (Chinese asparagus)	Root aqueous extract	Kun Ming mice	DPPH assay d-galactose induced mouse aging model Measurement of SOD, CAT, NOS, MDA, and NO contents Histopathology	Induced strong antioxidant activity Increased white blood cell count Improved SOD, CAT, and NOS activities in aging mice Increased NO content Reduced MDA content	[145]
*Asparagus officinalis* L. (asparagus)	Spear powder ethanol extract	Peripheral blood mononuclear cells (PBMCs)	2,2’-azinobis-(3-ethylbenzothiazoline-6-sulfonic acid) (ABTS) and ferric reducing ability of plasma (FRAP) assays Matrix metalloproteinase-1 (MMP-1) inhibitory activity Elastase inhibitory activity Hyaluronidase inhibitory activity Cytotoxicity effect	Inhibited the elevated levels of MMP-1, elastase, and hyaluronidase by 83.4 ± 1.5%, 70.4 ± 4.1%, and 75.2 ± 1.0%, respectively Presented an attractive source of natural anti-wrinkle ingredients	[146]
*Asparagus officinalis* L. (asparagus)	Aqueous stem extract	Normal human dermal fibroblasts	UV-B-irradiated NHDFs cells RT-PCR Western blot analysis Measurement of telomere length	Increased HSP70 mRNA levels in NHDFs Reduced HSP70 expression at mRNA and protein levels Preserved HSP70 quantity in UV-B-irradiated NHDFs Induced anti-photoaging effects by suppressing HSP70 expression in UV-irradiated dermal fibroblasts	[147]
**Amaranthaceae**					
*Amaranthus tricolor* L. (Chinese Spinach)	Red Spinach Extract Ointment	Wistar Rats	Measurement of collagen, elasticity, hydration, sebum, and pigment levels in animals	At 10%, increased skin hydration levels (64.84%) At 10%, increased skin collagen levels (56.25%) At 10%, increased skin elasticity levels (46.30%) At 10%, increased skin pigmentation levels (35.97%) At 10%, decreased sebum levels (40%)	[148]
**Asteraceae**					
*Cynara scolymus* L. (Artichoke)	Leaf extract	d-galactose-induced aging rats	Rat model of d-galactose-induced aging Measurement of hematological parameters Evaluation of SOD, GPx, and CAT activities, and MDA and LF (lipofuscin) levels in serum, liver, and brain	Increased SOD activity in brain, liver, and GSH-Px Decreased MDA content in serum, LF in brain and liverExhibited anti-aging proprieties	[149]
*Cynara scolymus* L. (Artichoke)	Bract aqueous extract	Immortalized human keratinocyte cell line (HaCaT)	High-performance liquid chromatography (RP-HPLC-DAD) DPPH assay Reactive oxygen species (ROS) scavenging activityIn vitro sun protection factor (SPF) measurement Human repeat insult patch testing (HRIPT) Chromameter evaluation of UV radiation-induced oxidative stress	Exhibited antioxidant and photoprotective activity	[150]
*Cynara scolymus* L. (Artichoke)	Leaf extract	Wistar rat	Determination of activities of enzymatic part of skin endogenous antioxidant defense system d-gal-induced aging changes in skin Total SOD activity and superoxide anion generation	Restored skin relative weight Decreased the generation rate of O_2_^•−^, H_2_O_2_, and LPx Activated the enzyme link in the innate antioxidant defense system in the d-galactosidase-induced skin aging model	[151]
*Cynara scolymus* L. (Artichoke)	Leaf extract	Wistar rats	Antioxidant capacity (AOC) determination Skin edema evaluation Total collagen (hydroxyproline) and hexosamine contents determination NF-κB determination	Restored skin relative weight Induced elevated solubility in neutral salt and acid Decreased pepsin solubility collagen fraction Restored the hexosamine/collagen ratio Decreased NF-κB activity Improved collagen metabolism Attenuated the progression of inflammation in the skin aging model	[152]
*Taraxacum officinale* F.H. Wigg. (Dandelion)	Aqueous extract	TM3 cells, an immature mouse Leydig cell line, and 18-week-old male Sprague Dawley rats	Western blot analysis Measurement of serum testosterone level Swimming retention test Measurement of sperm count and activity	Protected TM3 cells from serum restriction and oxidative stress via activation of ERK and Akt pathways Improved testosterone level and activation of spermatogenesis in rats Improved physical locomotion Improved quality of life for aging males	[153]
**Brassicaceae**					
*Brassica oleracea* L. var. *capitata* F. rubra (red cabbage)	Ethanol extract	Male Wistar mice	UV-B exposure Dermis-elastic fiber thickness assessment	No alteration in the thickness of the dermal layer following UV-B exposure	[154]
*Brassica oleracea* L. var. *italica* Plenck (*Broccoli*)	Ethanol extract of flowers	Normal human fibroblast cells	Ultraviolet irradiation RNA extraction RT-PCR ELISA	Decreased MMP-1 expression at both MMP-1 mRNA and MMP-1 protein expression Prevented UVB-induced MMP-1 expression at both mRNA and protein levels	[155]
*Raphanus sativus* L. (Radish)	Supercritical heat-treated radish skin and green extract	UV-induced Hos: HRM-2 wrinkled mouse	Evaluation of skin thickness, elasticity, and wrinkles induced by UVB lamp	Increased depth of wrinkles Increased expression of MMP-2 and MMP-2 genes Inhibited MMP-2 expression Improved skin wrinkles	[156]
**Cucurbitaceae**					
*Cucumis sativus* L. (Cucumber)	Juice	In vitro enzymatic assays	HPLC analysis DPPH assay Hyaluronidase inhibition assay Elastase inhibition assay	Exhibited DPPH free radical and superoxide radical scavenging activity Induced strong anti-hyaluronidase and anti-elastase activity	[157]
*Cucurbita moschata* Duchesne (pumpkin)	Seed petroleum ether extract	Fibroblast cell lines	Cytotoxicity assay SA-βgal (Senescence-Associated Beta Galactosidase) activity Molecular docking	Reduced the % of cell senescence in a dose-dependent manner	[158]
**Fabaceae**					
*Glycine max* (L.) Merr. (Soybean)	Protein hydrolysate extract	d-galactose-induced specific pathogen-free (SPF) Kunming mice	DPPH assay Hydroxyl radical scavenging activity (HRSA) Reducing power assay Characterization of peptides Evaluation of antioxidant activity (in vitro) of synthesized peptides	Reversed learning and memory impairments associated with aging Induced significant DPPH scavenging activity	[159]
*Glycine max* (L.) Merr. (Soybean)	Extract of (ethanol: hexane)	Male human volunteers	DPPH assay Anti-aging study (skin microrelief, skin elasticity, and skin capacitance)	Affected the skin elasticity and moisture contents Diminished skin scaliness, skin wrinkles, skin smoothness, and skin roughness Exerted potential skin anti-aging effects	[160]
*Glycine max* (L.) Merr. (Soybean)	Okara (soy pulp)	Male SAMP8 and senescence-accelerated resistant mouse 1 (SAMR1) mice	Barnes maze test Microbiota sequencing and analysis qRT-PCR Western blot analysis Immunohistochemical analysis Measurement of acetylcholine concentration	Decreased the inflammatory cytokine TNF-α Increased brain-derived neurotrophic factor (BDNF) Increased the expression of acetylcholine synthesis enzyme Increased the level of acetylcholine in the brain Prevented cognitive decline without dramatically altering the gut microbiome	[161]
*Glycine max* (L.) Merr. (Soybean)	Monascus-Fermented Soybean Extracts	In vitro assay of inhibitory enzyme activities	Tyrosinase inhibition assay Hyaluronidase inhibition assay Elastase inhibition assay Trolox equivalent antioxidant capacity (TEAC) assay	Increased antioxidant capacities depending on the dose Inhibited the activity of tyrosinase, hyaluronidase, and elastase Inhibited the activity of skin aging-related enzymes	[162]
*Glycine max* (L.) Merr. (Soybean)	Fermented soybean milk by *Lactobacillus plantarum*	Mice with premature aging induced by d-galactose	DPPH assay Histopathology Biochemical analysis of GSH, CAT, SOD, GPx, and MDA levels RT-PCR	Presented better ability to scavenge free radicals Protected the skin, spleen, and liver Reduced oxidative damage and inflammation Upregulated GSH, CAT, SOD, and GPx levels Decreased MDA content in liver, brain, and serum Improved antioxidant capacity in mice with d-galactose-induced premature aging	[163]
*Glycine max* (L.) Merr. (Soybean)	Black soybean peptides	Aging mice induced by d-galactose	Antioxidant activity assessment (in vivo)	Increased SOD and GPx activity in liver and serumReduced MDA contents in serumExhibited significant antioxidant activity in mice	[164]
*Vigna angularis* (Wild.) Ohwi and Ohashi (Red Bean)	Ethanolic extract	Human volunteers	Gel Formulation evaluation Anti-aging test (moisture level, skin pore size, evenness) Dark spot test	Improved moisture level, pore size, evenness, and number of black spots Transformed into a peel-off gel mask with anti-aging properties	[165]
*Phaseolus vulgaris* L. (Black Bean)	Phenolic extract	In vitro assay of inhibitory enzyme activities	Total phenolic content (TPC) Total anthocyanin content (ACN) Supercritical fluid (SCF) Leaching extractions DPPH and ABTS assays Tyrosinase inhibition assay Elastase inhibition assay	For the DPPH scavenging assay: IC_50_ = 0.32 ± 0.01 mg GAE/g coat For the ABTS assay: IC_50_ = 0.40 ± 0.03 mg GAE/g coatFor the tyrosinase enzymatic inhibition assay: IC_50_ = 10.44 ± 1.32 mg GAE/g coat For the collagenase enzymatic inhibition assay: IC_50_ = 8.33 ± 0.65 mg GAE/g coat For the elastase enzymatic inhibition assay: IC_50_ = 0.11 ± 0.02 mg GAE/g coat	[166]
*Vigna angularis* (Wild.) Ohwi and Ohashi (Azuki beans)	Aqueous extract	Normal human dermal fibroblasts cells and hairless mice	DPPH assay UV irradiation ROS assay MTT assay Topical application Wrinkle measurement Measurement of physiological skin functions Histopathology Western blot analysis	Induced antioxidant activity in UVB-exposed human dermal fibroblasts Suppressed MMP-1 production (90%) Suppressed wrinkle formation and skin thickness Prevented skin photoaging accelerated by UVB radiation	[167]
**Malvaceae**					
*Abelmoschus* *Esculentus* (L.) Moench (Okra)	Ethanol extract	Human neuroblastoma (SK-N-SH) cell lines	Cell viability assay ROS assay SA-β-galactosidase enzyme assay	Promoted cell viability over reduced ROS content and SA-β-gal positive cells Developed synaptic plasticity by inhibiting AChE activity Attenuated the negative responses of aging neurons	[168]
**Polygonaceae**					
*Rheum rhabarbarum* L. (Rhubarb)	Rhubarb Preparation	Mice with cerebral malfunction induced by d-galactose	Determination of Ach and AchE levels Assessment of peroxidase level	Increased cortical CAT and GPx activities Decreased AchE activity and increased Ach level Increased cerebellar SOD, CuSOD, and Mn-SOD activities Lowered LPO level Increased cortical CAT activity Decreased the level of cerebellar LPO Improved memory with its ability Regulated the activities of CAT, GSH-px, and SOD Inhibited the activity of AchE Increased Ach level	[169]
*Rumex crispus* L. (Curly Dock)	Roots and leaves hydroethanolic extract	In vitro assay of inhibitory enzyme activities	Measurement of UV absorption DPPH and ABTS assays NO assay Phosphomolibdate assay MMP-1, MMP-8, and MMP-13 inhibitor screening tests	Exhibited the highest inhibitory effect on all MMP enzymes Presented high UV protection Exhibited strong antioxidant capabilities	[170]
**Apiaceae**					
*Daucus carota* L.	Seeds ethanol and petroleum ether extracts	Sprague Dawley rats’ brain aging induced by d-galactose	Carrot seed oil fatty acid assessment Anti-aging assessment	Removed both CAT reduction and MDA elevation Exhibited remarkable antioxidant and anti-inflammatory properties	[171]

**Table 2:** Anti-aging effects of natural molecules isolated from vegetables.

Molecules (Origin)	Models Used	Methods	Key Results	References
Allicin (*Allium sativum*)	In silico molecular docking	Molecular docking	Presented the highest potential against premature aging Inhibited leukocyte elastase activity	[172]
Caffeic acid, S-allyl cysteine, and uracil (*Allium sativum*)	HR1 hairless mouse	Masson’s trichrome staining ROS assay Western blot analysis	Inhibited the degradation of y-type procollagen Inhibited the expression of matrix metalloproteinases in vivo Improved the histological disorder of collagen fibers and oxidative stress in vivo Decreased oxidative stress and inflammation via modulation of NF-κB and AP-1 activities	[173]
Glucosinolate (glucoraphanin and sulforaphane) and phenolics (kaempferol and quercetin) from broccoli (*Brassica oleracea*)	In silico molecular docking	Drug-likeness and bioactivity prediction Biological activity prediction using PASS online	Presented a strong interaction with Keap1 Inhibited Keap1-Nrf2 interaction Enhanced HMOXI expression Inhibited peroxidase	[174]
Glycoprotein (*Daucus carota*)	Fibroblasts of the dermis of the human body	DPPH assy Inhibition of lipid peroxidation Collagen type-1 creation promotion experiments Inhibition of MMP-1 expression	Exhibited good antioxidant activity Expressed high lipid peroxidation Promoted the generation of collagen type-1 Reduced the MMP-1 Eliminated ROS Acted as an antioxidant and anti-aging agent in skin exposed to solar ultraviolet light	[175]
Water-soluble protein (*Vicia faba*)	Human fibroblasts (TIG-1)	Effect of water-soluble protein against antioxidant enzyme activities and GSH concentration Biochemical analysis.	Decreased cytosolic SOD activity Presented elevated CAT activity in young and old cells	[176]
Arctigenin, matairesinol, arctiin, (iso) lappaol A, lappaol C, and lappaol F (*Arctium lappa* L.)	*Caenorhabditis elegans*	DPPH assay ROS assay Lifespan assay juglone-induced oxidative stress assay RT-PCR	Exhibited good antioxidant activity (strongest observed with matairesinol) Extended the average lifespan of *C. elegans*	[177]
Dioscin, allantoin, and diosgenin (*Dioscoreae Rhizoma*)	molecular docking	Constructing the protein–protein interaction (PPI) network Molecular docking Gene ontology (GO) functional enrichment analysis Kyoto encyclopedia of genes and genomes (KEGG) pathway enrichment analysis	Regulated the expression of target proteins via enriched signaling pathways Inhibited tumor proliferation and metastasis Regulated metabolism Promoted nerve repair by regulating the expression of targets (MAPK3, HADC3, HADC1, RXRA, STAT3, etc.)	[178]
Trp-Pro-Lys (WPK) and Ala-Tyr-Leu-His (AYLH) *Glycine max* (Soybean)	d-galactose-induced specific pathogen-free (SPF) Kunming mice	Shuttle box test Biochemical analysis DPPH assay HRSA Reducing power assay Separation and purification of peptides.	Attenuated H_2_O_2_-induced oxidative damage in PC12 cells Prevented age-related learning and memory impairments and oxidative stress	[159]

## Data Availability

Not applicable.

## Abbreviations

ABTS	2,2’-Azinobis (3-ethylbenzothiazoline-6-sulfonic acid
ACN	Anthocyanin
AMPK	AMP-Activated Protein Kinase
AOC	Antioxidant Capacity
AT	Ataxia-Telangiectasia
CAT	Catalase
COX-2	Cyclooxygenase-2
Cu/Zn-SOD	Cupro-Zinc Superoxide Dismutase
CYP	Chinese Yam Polysaccharides
DNA	Deoxyribonucleic Acid
DDR	DNA Damage Response
DPPH	1,1-Diphenyl-2-Picrylhydrazyl
ECM	Extracellular Matrix
EXO1	Exonuclease 1
FRAP	Ferric Reducing Antioxidant Power
FT-IR	Fourier Transform Infrared
GO	Gene Ontology
GPXs	Glutathione Peroxidase
GSH	Glutathione
HDL	High-Density Lipoprotein
HRIPT	Human Repeat Insult Patch Testing
HPLC-MS	High-Performance Liquid Chromatography–Mass Spectrometry
HRSA	Hydroxyl Radical Scavenging Activity
IC_50_	50% Inhibitory Concentration
IL-1	Interleukin-1
iNOS	Inducible Nitric Oxide Synthase
KEGG	Kyoto Encyclopedia of Genes and Genomes
LDL	Low-Density Lipoprotein
LF	Lipofuscin
MIC	Minimum Inhibitory Concentration
mtDNA	Mitochondrial DNA
MMPs	Matrix Metallo-Proteinases
Mn-SOD	Manganese Superoxide Dismutase
mRNA	Messenger RNA
NAD	Nicotinamide Adenine Dinucleotide
NF-κB	Nuclear Factor-κB
NO	Nitric Oxide
NOS	Nitric Oxide Synthase
ORAC	Oxygen Radical Absorbance Capacity
OS	Oxidative Stress
PMS2	Post-Meiotic Segregation increased 2
PPI	Protein–Protein Interaction
RNA	Ribonucleic Acid
ROS	Reactive Oxygen Species
RP-HPLC-DAD	Reversed-Phase High-Performance Liquid Chromatography with a Diode Array Detector
RT-PCR	Reverse Transcription Polymerase Chain Reaction
SA-gal	Senescence-Associated Galactosidase
SCF	Supercritical Fluid
SOD	Superoxide Dismutase
SPF	Sun Protection Factor
TBARS	Thiobarbituric Acid Reactive Substances
TC-NER	Transcription-Coupled Nucleotide Excision Repair
TEAC	Trolox Equivalent Antioxidant Capacity
TNF-α	Tumor Necrosis Factor-A
TPC	Total Phenolic Content
UVB	Ultraviolet B
WRN	Werner’s Syndrome Protein
WSP	Water Soluble Protein
